# Effects of High-Frequency Repetitive Transcranial Magnetic Stimulation Combined with Motor Learning on Motor Function and Grip Force of the Upper Limbs and Activities of Daily Living in Patients with a Subacute Stroke

**DOI:** 10.3390/ijerph20126093

**Published:** 2023-06-09

**Authors:** Jungwoo Shim, Seungwon Lee

**Affiliations:** 1 Department of Rehabilitation Medicine, Chungnam National University Sejong Hospital, Sejong-si 30099, Republic of Korea; sjw0812@cnuh.co.kr; 2 Department of Physical Therapy, Sahmyook University, Seoul 01792, Republic of Korea

**Keywords:** repetitive transcranial magnetic stimulation, motor learning, upper-limb motor function, grip force, stroke

## Abstract

Functional paralysis of the upper extremities occurs in >70% of all patients after having a stroke, and >60% showed decreased hand dexterity. A total of 30 patients with a subacute stroke were randomly allocated to either high-frequency repetitive transcranial magnetic stimulation combined with motor learning (*n* = 14) or sham repetitive transcranial magnetic stimulation combined with motor learning (*n* = 16). High-frequency repetitive transcranial magnetic stimulation combined with the motor learning group was conducted for 20 min (10 min of high-frequency repetitive transcranial magnetic stimulation and 10 min of motor learning) three times a week for 4 weeks. The sham repetitive transcranial magnetic stimulation combined with the motor learning group received 12 20-min sessions (10 min of sham repetitive transcranial magnetic stimulation and 10 min of motor learning). This was held three times a week for 4 weeks. Upper-limb function (Fugl-Meyer Assessment of the Upper Limbs) and upper-limb dexterity (box and block tests) concerning upper-limb motor function and grip force (hand grip dynamometer), and activities of daily living (Korean version of the modified Barthel index), were measured pre- and post-intervention. In both groups, there were significant improvements in the upper-limb motor function, grip force, and activities of daily living (*p* < 0.05). Regarding grip force, the high-frequency repetitive transcranial magnetic stimulation combined with the motor learning group improved significantly compared to the sham repetitive transcranial magnetic stimulation combined with the motor learning group (*p* < 0.05). However, except for grip force, there were no significant differences in the upper-limb motor function or activities of daily living between the groups. These findings suggest that high-frequency repetitive transcranial magnetic stimulation combined with motor learning is more likely to improve grip force than motor learning alone.

## 1. Introduction

A stroke is the sudden onset of neurological symptoms caused by cerebrovascular injury, and most adult patients with a stroke experience hemiplegia. After a stroke, 10–12% of all patients die, and more than 50% of survivors have long-term disability and daily life problems [1]. Regarding motor ability and functional level, many patients recover within 3 months of onset and show progressive recovery at 3–6 months [2]. Therefore, performing activities of daily living and healing the upper-limb function during early attentional treatment are major significant after a stroke [3].

These strokes cause motor dysfunction on the paralyzed side, indicating abnormalities in balance, asymmetrical postures, and symptoms of dysfunction of the upper extremities and hands [4]. In particular, functional paralysis of the upper extremities occurs in more than 70% of all patients after stroke, and more than 60% show decreased hand dexterity [5]. Additionally, when comparing grip force and upper-limb function measurements in patients with a subacute stroke, it has been observed that failure of early grip recovery is associated with impaired upper-limb function [6]. For patients with a stroke, activities of daily living recovery are essential for the rehabilitation purpose of returning patients with a stroke to normal life. Further, incorporating all remaining abilities to overcome their disability helps them achieve mental and social satisfaction and physical recovery [7]. Early recovery of upper-limb movement function, grip strength, and activities of daily living is vital for patients with a stroke [8].

The motor learning (ML) principle is used as a treatment for post-stroke dysfunction [9,10]. Some studies have compared the Bobath concept, neurodevelopmental therapy, and motor learning applications [10,11], still widely used to treat mild and severe upper-limb movements, with constraint-induced movement therapy on the less affected side. These treatments showed positive results in most studies but limited normal functional recovery in stroke survivors, with statistically significant results but very low effect sizes.

Moreover, repetitive transcranial magnetic stimulation (rTMS) has recently been widely used to restore upper-limb motor function in patients with a stroke. rTMS is a non-invasive treatment used to control the excitation of the cortex. Transcranial magnetic stimulation depolarizes nerve cells under a coil by applying strong electrical stimulation to the electromagnetic coil, resulting in changes in cerebral cortex excitation according to intensity, frequency, and the total number of stimuli [12,13]. Previous studies have shown that rTMS is effective for grip strength in patients with subcortical region damage and brain strokes [14] and for upper-limb motion function through rTMS and occupational therapy [15]. Although rTMS is believed to be effective when combined with other treatments, few studies have been conducted to confirm this hypothesis.

Furthermore, a previous study showed that high-frequency repetitive transcranial magnetic stimulation (HF-rTMS) is more effective than low-frequency rTMS [16]. However, studies on HF-rTMS and upper-limb exercise are insufficient, most of which have combined low-frequency repetitive transcranial magnetic stimulation and upper-limb exercise. According to McCabe et al. [17], motor learning is more effective when combined with other treatments or techniques such as robot training and functional electrical stimulation. However, the only study that combined motor learning with rTMS was a comparative study involving healthy adults.

Therefore, this study aimed to test the hypothesis that the combination of HF-rTMS and motor learning would result in greater improvements in upper-limb function compared to sham stimulation and motor learning alone in patients with a subacute stroke. This study aimed to present the effect of a program combining HF-rTMS with motor learning on motor function, grip force of the upper limbs, and activities of daily living in this patient population.

## 2. Methods

### 2.1. Participants

The study participants were inpatients with a subacute stroke who had received physical therapy at C University Hospital in Daejeon City. The inclusion criteria were as follows: (1) hemiplegia due to stroke, (2) factors within 6 months of onset, (3) subcortex damage through diagnoses of magnetic resonance imaging, (4) motor defects in the damaged upper extremities, and (5) a Mini-Mental State Examination score greater than 24 points. The exclusion criteria were as follows: (1) permanent damage such as arrhythmia; (2) upper-limb fractures; (3) neurological damage such as Parkinson’s disease; (4) multiple sclerosis; (5) other reasons limiting upper-limb movement; (6) epilepsy or family history of epilepsy; (7) wearing a metal tube in the skull or pacemaker; or (8) lesions in the occipital lobe [18]. Prior to the experiment, the study’s purpose and procedures were thoroughly explained to the participants, emphasizing their right to withdraw at any point, even at the beginning and throughout the study period. Only those who consented to participate were recruited, and written informed consent was obtained from all participants.

### 2.2. Sample Size Calculation

**Figure 1:**
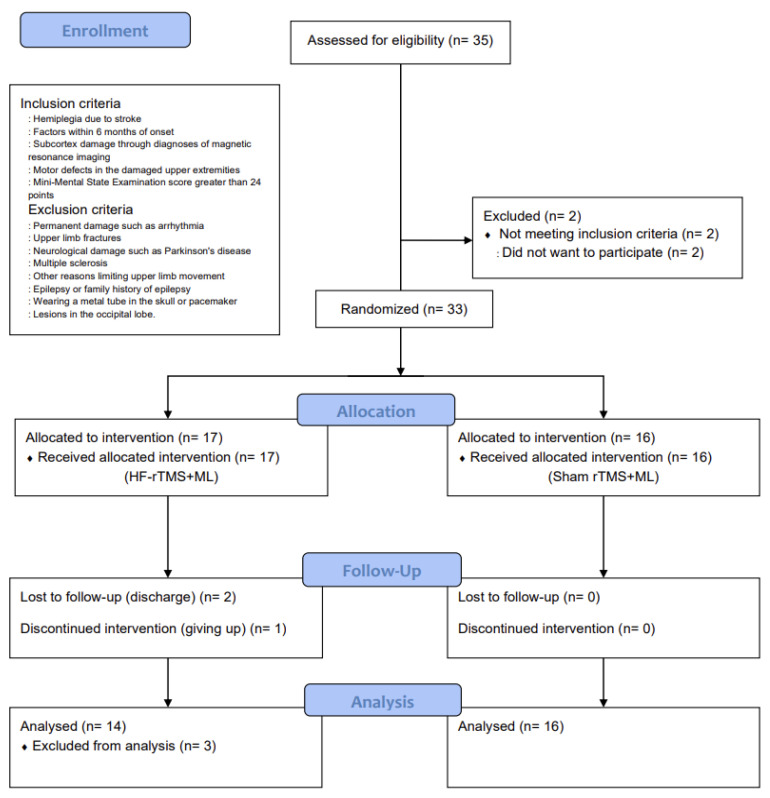
CONSORT flow diagram depicting the flow of participants through the study.

The number of participants required for the study was calculated using G*Power 3.1.9.2 [19]. The significance level was set at 0.05, and the effect size was set at 1.12, which was based on the average treatment effect size value of Fugl-Meyer Assessment of the Upper Limbs (FMA-U/L) that outcome measure of upper-limb motor function in previous studies that improved the upper-limb motor function of patients with a stroke by combining rTMS and upper-limb exercises [20]. The number of samples to maintain the power of 0.80, 14 per group, required a total of 28 people. However, 35 participants were selected in anticipation of a 20% dropout rate (Figure 1).

### 2.3. Study Design

This was a randomized, sham-controlled, double-blind trial with a two-group design. The study group was randomly selected using a simple random sampling method and was classified into a high-frequency repetitive transcranial magnetic stimulation combined with the motor learning group (HF-rTMS + ML group) and a sham repetitive transcranial magnetic stimulation combined with the motor learning group (sham rTMS + ML group) before the intervention. This study was approved by the Institutional Review Board of Sahmyook University (2-1040781-AB-N-01-2016071HR). This trial has been registered at ClinicalTrials.gov (registration number: NCT05176613). A total of 35 participants were recruited, and 33 were selected based on the inclusion and exclusion criteria. They were then assigned to either the HF-rTMS + ML group (*n* = 17) or the sham rTMS + ML group (*n* = 16) through random placement in Microsoft Excel. In the HF-rTMS + ML group, 2 patients were excluded due to discharge, and 1 dropped out of the intervention (*n* = 1), leaving 30 participants in the study (Figure 1).

### 2.4. Intervention

In the HF-rTMS + ML group, high-frequency repetitive transcranial magnetic stimulation and motor learning were combined, and in the sham rTMS + ML group, sham rTMS and motor learning were combined. The intervention method of the HF-rTMS combined with motor learning group was HF-rTMS stimulated a 70-mm, 8-shaped coil stimulator of the Magstim Company (Whitland, UK) on the damaged cerebral cortex. Prior to the application of HF-rTMS, the motor point that stimulated the maximum thresholds on the primary motor cortex, causing flexion of the oppositely affected index finger, was identified. If the cerebral hemisphere did not show a kinetic response, even at the maximum stimulus, the motor point of the opposite hemisphere changed symmetrically. The intensity of the stimulus was 80% of the resting motor threshold, which means that the motor-evoked potential above the first dorsal interosseous muscle can produce 50 μV (more than 5 out of 10 stimuli). Participants sat on a chair holding their heads. Stimulation was conducted at a high frequency (10 Hz) for 2 s, and the rest was conducted for 58 s for a total of 200 times for 10 min on the affected hemisphere [16,21].

**Figure 2:**
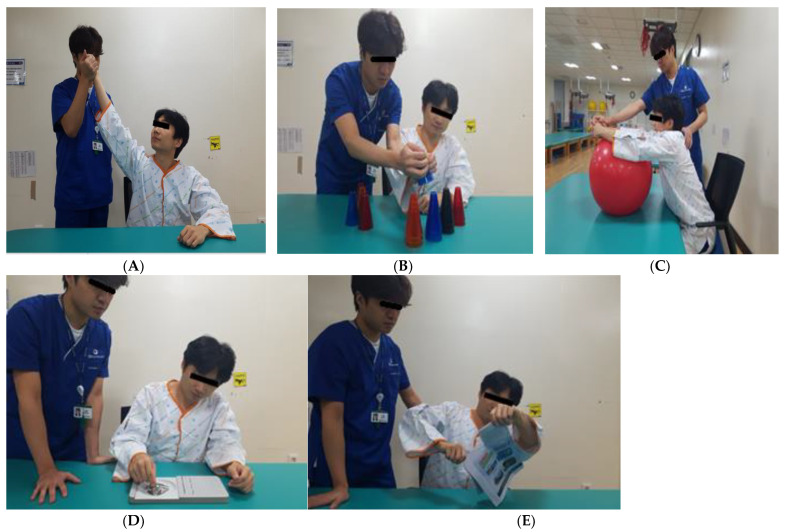
Motor learning (ML). (**A**) External rotation training. (**B**) Stacking cups by transferring. (**C**) Pushing and pulling the ball. (**D**) Inserting and removing pegs. (**E**) Tearing a newspaper.

Motor learning was conducted for 2 min each over five rounds (Figure 2). The first involved external rotation training to the maximum shoulder joint range in the sitting position. The participant held the affected elbow joint such that the center of mass could be stretched as much as possible in the sitting position. The second method involved stacking cups by transferring them from the unaffected side to the affected side and by transferring twenty-five plastic cups of five colors while sitting with both hands inserted in the target to stack them in the direction of the damage. Third, by pushing and pulling the ball forward and backward with the hands folded, the participant placed a 55 cm healing ball on the table in a sitting position and pushed and pulled it forward with the upper limbs. The fourth method involved inserting and removing pegs from the pegboard, and the subject used the affected hand in a sitting position to place pegs into the outer grip of the fingers and then took them out and placed them in the basket on the affected side. The fifth method involved tearing a newspaper. Sitting at a table, the participant held the newspaper with the unaffected hand and tore it with the affected hand [20]. The intervention was conducted for 20 min (10 min of HF-rTMS and 10 min of motor learning) three times a week for 4 weeks.

In the sham rTMS + ML group, sham rTMS provided a small intensity of 2% of the resting motor threshold that could not cause excitement in the motor cortex but was set to the same frequency of noise as the HF-rTMS, and motor learning was applied equally. The intervention was conducted for 20 min (10 min of sham HF-rTMS and 10 min of motor learning) three times a week for 4 weeks [22].

### 2.5. Functional Evaluation

The Fugl-Meyer Assessment of the Upper Limbs (FMA-U/L) was used to evaluate upper-limb functionality. The FMA-U/L score is a tool for evaluating the body’s structure, function, and activity level as an evaluation tool for evaluating motor function according to Brunnstrom’s recovery phase in Step 6 [23]. The FMA-U/L is the most commonly used evaluation tool for upper-limb evaluation and consists of the sum of shoulder/elbow movement and wrist/hand items [17]. There are 33 evaluation items related to the upper limbs, with a perfect score of 66 and a high level of intra-rater reliability (range of 0.95–1.0) [24]. The inter-rater reliability between the assessor and therapist is also highly valued, with an 0.98 exercise score and a 0.93 sensory score (0–2; maximum = 66) [25]. 

Box and block tests (BBT) assessed upper-limb dexterity. The BBT consists of cubical pieces of wood 2.54 cm long and a rectangular box with a partition in the center 53.7 × 8.5 × 27.4 cm in size. The intra-rater reliability of the BBT is 0.96, and the inter-rater reliability is 0.99 [26].

A force gauge (300 lb hydraulic digital hand dynamometer with LCD, Base-line Evaluation Instruments, New York, NY, USA) was used to measure grip force. Seated in a chair, the participants attached their elbows to the trunk, maintained 90 degrees in the elbow joint, and remained neutral in the forearm and wrist position. To obtain the maximum value, the number of times the hand was applied was measured thrice for each participant [25]. 

The Korean version of the modified Barthel index (K-MBI) was used to evaluate activities of daily living. The K-MBI consists of seven personal handling items and ambulation and three items related to personal hygiene, bathing, eating, toilet treatments, climbing stairs, dressing, bowel control, walking, wheelchairs, artificial limbs, and moving beds. The evaluation method involves instructing the participants on each item and observing their responses. Each action was scored on a five-point scale, with a perfect score of 100 points, 0–20 points for complete dependence, 21–61 points for heavy dependence, 62–90 for moderate dependence, 91–99 for mild dependence, and 100 points for full independence. The intra-rater reliability was 0.87, and the inter-rater reliability was 0.93 [27]. All evaluations were performed by the same physical therapist before and after the intervention.

### 2.6. Data Analysis

All statistical analyses were performed using SPSS ver. 25.0 (IBM Co., Armonk, NY, USA) to calculate means and standard deviations. An independent *t*-test was used to test homogeneity, and the Shapiro–Wilk test was used to test normality. Paired *t*-tests were conducted to measure the changes after the intervention in the HF-rTMS + ML and sham rTMS + ML groups, and they were analyzed using a two-way repeated-measures analysis of variance to confirm the interactions between factors (time × group). All statistical significance levels were set at *p* < 0.05.

## 3. Results

The demographic characteristics of the participants, including sex, age, height, weight, affected side, onset period, and MMSE-K score, did not differ significantly between the HF-rTMS + ML and sham rTMS + ML groups (Table 1). 

**Figure 3:**
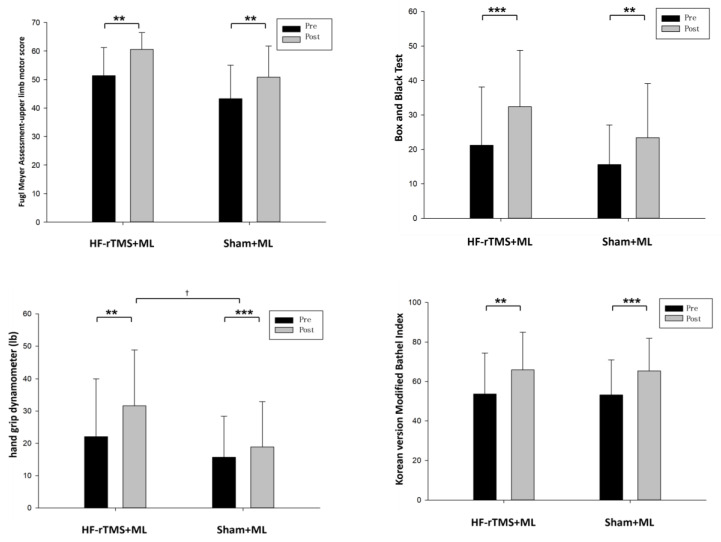
Change in upper-limb function according to each group. ** *p* < 0.01, *** *p* < 0.001 is the comparison before and after the experiment, and † *p* < 0.05 is the comparison between groups.

**Figure 4:**
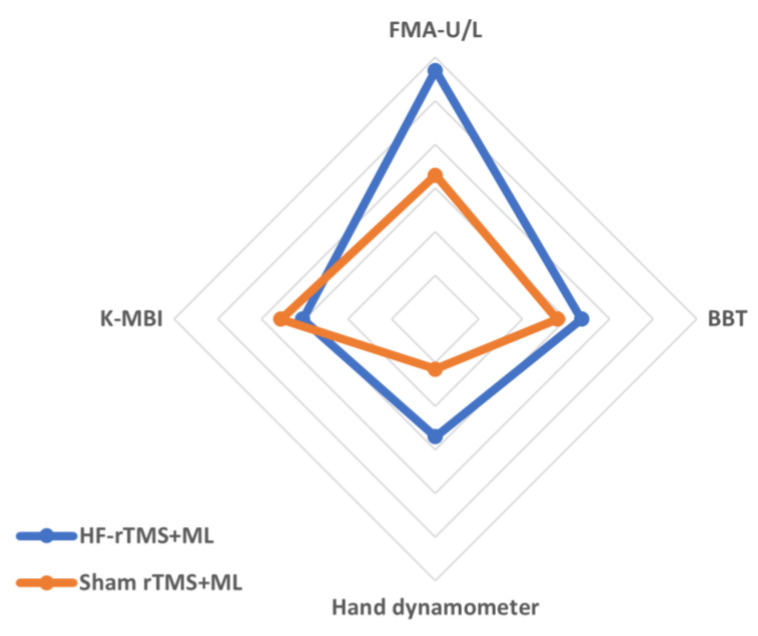
Comparisons of the effects of intervention methods based on Cohen’s d effect sizes. Values are the effect sizes of the intervention methods using Cohen’s d.

The changes in the group’s upper-limb motor function after the experiment were as follows: in terms of upper-limb function, the HF-rTMS + ML group increased significantly from 51.35 to 60.57 after the intervention (*p* < 0.05), and the sham rTMS + ML group increased statistically significantly from 43.25 to 50.81 after the intervention (*p* < 0.05). However, the two groups had no significant differences before and after the intervention (Figure 3, Table 2). When the intervention effects of each group were compared numerically using Cohen’s d, the most significant effect was observed in the HF-rTMS + ML group (d = 1.14) (Figure 4, Table 2).

Upper-limb dexterity in the HF-rTMS + ML group increased statistically significantly from 21.21 before the intervention to 32.43 after the intervention (*p* < 0.05), while that in the sham rTMS + ML group increased statistically significantly from 15.63 before the intervention to 23.44 after the intervention (*p* < 0.05). However, the two groups had no significant differences before and after the intervention (Figure 3, Table 2). When the intervention effects of each group were compared numerically using Cohen’s d, the most considerable effect was observed in the HF-rTMS + ML group (d = 0.67) (Figure 4, Table 2).

The changes in the group’s grip force before and after the experiment are as follows (Table 2): for grip force, the HF-rTMS + ML group increased statistically significantly from 22.07 before the intervention to 31.64 after the intervention (*p* < 0.05), and the sham rTMS + ML group increased statistically significantly from 15.68 before the intervention to 18.87 after the intervention (*p* < 0.05). There was also a significant difference between the two groups before and after the intervention (*p* < 0.05) (Figure 3, Table 2). When the intervention effects of each group were compared numerically using Cohen’s d, the most significant effect was observed in the HF-rTMS + ML group (d = 0.54) (Figure 4, Table 2). 

In activities of daily living, the HF-rTMS + ML group showed a statistically significant increase, from 53.71 before the intervention to 65.85 after the intervention (*p* < 0.05). Similarly, the sham rTMS + ML group showed a statistically significant increase from 53.18 before the intervention to 65.37 after the intervention (*p* < 0.05). However, the two groups had no significant differences before and after the intervention (Figure 3, Table 2). When the intervention effect of each group was compared numerically using Cohen’s d, the most considerable effect was observed in the sham rTMS + ML group (d = 0.71) (Figure 4, Table 2).

## 4. Discussion

rTMS has different effects depending on the stimulation frequency, intensity, and time, and there is a known risk of side effects, such as seizures, in healthy people if applied for more extended periods above motor thresholds [28]. In particular, while high-frequency rTMS is known to be more dangerous than low-frequency rTMS, rTMS with a frequency of 3 or 10 Hz is considered to be safe [29]. Through many interventions, previous studies have proven the effect of upper-limb treatment in stroke patients [30,31,32,33,34,35,36]. To verify the maximum common effect within this safety range, this study aims to ascertain the impact of motor function and grip force of the upper limbs and activities of daily living by means of an intervention with 10 Hz high-frequency rTMS and motor learning in subacute patients with a stroke. The present study investigates significant differences in upper-limb motor function, upper-limb dexterity, grip force, and activities of daily living, pre- and post-intervention, in both groups. Nonetheless, no difference between groups was found except for grip force. This was consistent with previous studies, such as that of Higgins et al. [37], which also combined rTMS and task-oriented training for upper-limb rehabilitation in post-stroke patients. In their study, the group that received actual rTMS and task-oriented training showed significant improvement (*p* < 0.05) in upper-limb function, grip strength, and finger pinch strength compared to the group that received sham rTMS and task-oriented training, as measured by the Wolf Motor Function Test (WMFT) after 4 weeks of intervention. However, there were no significant differences between the two groups. Similarly, in the study by Harvey et al. [20], which also combined rTMS and task-oriented training, there was no significant difference in the FMA-U/L scores between the group that received actual rTMS and task-oriented training and the group that received sham rTMS and task-oriented training after 4 weeks of intervention, as observed in this study. In the analysis of the differences between the two groups with the standardized mean difference using the effect size, Cohen’s d values were confirmed to be small. However, FMA and grip force were larger in the HF-rTMS + ML group than in the sham + rTMS group [38]. This demonstrates that rTMS and motor learning are intervention methods that can improve the motor function and grip force of the upper limbs and the performance of activities of daily living in patients with a stroke. Furthermore, both intervention methods were highly effective in improving motor function, grip force of the upper limbs, and performance of activities of daily living. It is thought that the motor learning method will significantly improve these functions.

This study only showed a difference between the groups’ grip force, which can be explained by the fact that rTMS has a more significant effect on improving grip force than on the recovery of upper-limb motor function. This is consistent with several previous studies examining hand function, grip force, and finger grip using rTMS [39,40,41]. In addition, in several studies, high-frequency rTMS improved grip force and typing ability compared with low-frequency rTMS [25,37]. This shows the effect of rTMS on the grip force of the affected hand in stroke patients, and it can be predicted that the impact will be more significant with high-frequency rTMS than with low-frequency rTMS. However, because the characteristics of patients with a stroke and the degree of recovery at each stage are different, the generalizability of these results is limited. 

Hand grip force has been used as a predictor of functional independence and motor performance in patients with a subacute stroke [6,42]. Recovery of grip force is an essential indicator of the restoration of upper-limb motor function and is directly proportional to the ability of the upper limbs to perform complex motor tasks. This is particularly relevant to patients with a stroke and has been widely reported in previous studies [42]. In this study, high-frequency rTMS improved grip force more significantly than upper-limb motor function, suggesting the potential clinical relevance of this treatment approach. Moreover, this study offers novelty compared to previous research by employing a motor learning method based on the Bobath concept, which is specifically tailored for enhancing upper-limb motor function. In contrast to conventional interventions such as occupational therapy [15], constraint-induced movement therapy [30], or task-oriented therapy [37], this study introduced a unique intervention combining the Bobath-based motor learning method with rTMS. The results demonstrated significant improvements in upper-limb motor function, grip strength, and activities of daily living. These findings highlight the innovative nature of this study in comparison to previous research in the field. A major limitation of this study was that the participants were patients with a subacute stroke whose maximum recovery was achieved after brain damage [2]. Natural recovery due to the plasticity of brain tissue was excluded and compared with that of the control group. Based on these results, research is needed to compare not only subacute patients with a stroke but also repeated measurements at various stages after the stroke and interventions longer than 4 weeks to confirm the effect on upper-limb motor function and grip. 

## 5. Conclusions

Our study demonstrated that the combination of HF-rTMS and motor learning is a promising intervention for improving upper-limb motor function, grip force, and activities of daily living in patients with a subacute stroke. This is an important finding, as it suggests that HF-rTMS could be a useful addition to current physical therapy for stroke patients. Furthermore, our study adds to the literature by showing that HF-rTMS is particularly effective in improving grip force, which has important implications for patients’ ability to perform activities of daily living. However, further research is required to understand the optimal HF-rTMS parameters for motor recovery fully. Overall, our study contributes to the development of evidence-based interventions for stroke rehabilitation and provides a foundation for future research in this area.

## Figures and Tables

**Table 1:** General and medical characteristics and homogeneity tests of the study participants.

	HF-rTMS + ML	Sham rTMS + ML	*t* (*p*) ^a^
Sex (M/F)	7/7	11/5	1.03 (0.31)
Age (years)	67.28 ± 10.80	63.56 ± 16.09	0.73 (0.47)
Height (cm)	161.30 ± 9.20	164.15 ± 10.42	−0.79 (0.44)
Weight (kg)	58.95 ± 9.52	63.57 ± 9.51	−1.33 (0.19)
Affected side (Left/Right)	7/7	8/8	0.00 (1.00)
On set (month)	3.57 ± 1.39	3.06 ± 1.56	0.93 (0.36)
MMSE-K (score)	27.71 ± 2.46	26.93 ± 2.32	0.90 (0.38)

Values are mean ± SD unless otherwise indicated. HF-rTMS, high-frequency repetitive transcranial magnetic stimulation; ML, motor learning; MMSE-K, Korean version of the Mini-Mental State Examination. ^a^ *t*-value (*p*-value).

**Table 2:** Change in upper-limb motor function, grip force, and activities of daily living according to each group.

Variable	HF-rTMS + ML		ES ^a^	Sham rTMS + ML		ES ^a^	Time × Group	
	Pre-Test	Post-Test		Pre-Test	Post-Test		*F*	*p*
Upper-limb motor function								
FMA-U/L	51.35± 9.82	60.57± 5.84 ^b^	1.14	43.25 ± 11.79	50.81 ± 10.91 ^b^	0.66	0.29	0.59
BBT	21.21 ± 16.93	32.43 ± 16.38 ^b^	0.67	15.63 ± 11.53	23.44 ± 15.72 ^b^	0.56	1.31	0.26
Grip force								
Hand dynamometer	22.07 ± 17.92	31.64 ± 17.17 ^b^	0.54	15.68 ± 12.71	18.87 ± 14.07 ^b^	0.23	9.16	0.00 ^c^
Activities of daily living								
K-MBI	53.71 ± 20.66	65.85 ± 19.07 ^b^	0.61	53.18 ± 17.76	65.37 ± 16.50 ^b^	0.71	0.00	0.91

Values are mean ± SD unless otherwise indicated. FMA-U/L, Fugl-Myer assessment of upper limbs; BBT, Box and Block Tests; K-MBI, the Korean version of the modified Bathel Index. ^a^ Effect size using Cohen’s d. ^b^ Paired *t*-test, *p* < 0.05, compared with a pre-test. ^c^ Two-way analysis of variance, *p* < 0.05, compared with the time × group.

## Data Availability

Not applicable.
